# Promoting Psychological Well-Being at Workplace through Protean Career Attitude: Dual Mediating Effect of Career Satisfaction and Career Commitment

**DOI:** 10.3390/ijerph191811528

**Published:** 2022-09-13

**Authors:** Jun-Chul Ha, Jun-Woo Lee

**Affiliations:** 1 Department of Electrical System Engineering, Hanbat National University, Daejeon 34158, Korea; 2 Department of Business Administration, Hanbat National University, Daejeon 34158, Korea

**Keywords:** protean career attitude, psychological well-being, career satisfaction, career commitment, competitive advantage

## Abstract

The management paradigm of SMEs is changing due to the recent Fourth Industrial Revolution and the changing COVID-19 environment. To respond to these changes, companies are focusing on protean career attitude (PCA) and psychological well-being (PWB) of employees to improve corporate performance. Under these circumstances, this study investigated the structural relationship of the dual mediation effects of career commitment and career satisfaction in the relationship between PCA and PWB. To this end, this study targeted 307 employees of Korean small and medium-sized enterprises (SMEs), and the results are as follows. First, PCA was found to have a positive effect on career satisfaction and PCA was found to have a positive effect on career commitment. Second, PCA was found to have a significant effect on PWB. Third, career satisfaction, a parameter of this study, was found to have a positive impact on career commitment; in the relationship between PCA and PWB, the dual mediation effects of career satisfaction and career commitment were found to be significant. Finally, this study provided discussions and theoretical and practical implications based on those results, as well as directions for future research.

## 1. Introduction

Along with the advent of the Fourth Industrial Revolution, innovative changes are recently occurring in the whole of society in line with the emergence of new industries and rapid changes in business environments. These changes have caused instability in the job market and flexibility in the labor market, which have also affected the changes in the career paradigm [1].

In traditional career management, companies took the lead in career development, but due to downsized organizations, stagnant positions, and a preference for experts for better performance, companies have faced a realistic limit in taking responsibility for the career development of their employees. As such, the emergence of new industrial environments has emphasized the importance of self-directed career development in improving corporate performance and securing a source of sustainable growth [2,3,4].

In this rapidly changing environment, the era has come when individuals must actively adapt to external environmental changes and take responsibility for their own career development. In this environment where employability becomes more important, individuals become a subject that actively strives to secure their own employability, not just a passive entity belonging to an organization or company; in order to secure high employability and adapt to changing environments, it became more important for individuals to develop necessary capacities [5]. From a corporate point of view, protean career attitude (PCA) has become more important, as it contributes to changes in individual interests, abilities, and values according to the organizational context, enabling employees to grow through new experiences and learning, and changing the interests, abilities, and values of employees in the organizational context [6,7].

Therefore, it is necessary to pay attention to the PCA, which includes the individual’s self-directed career choices and value-driven characteristics. PCA is an attitude by which an individual develops a career based on his/her own values [1]. It is an individual’s attitude by which, based on their values, the individual takes responsibility for their career plans and management by themselves, and manages their careers [8]. Through PCA, individuals can achieve high work performance by improving their subjective success and self-efficacy through self-directed career management.

Meanwhile, psychological well-being (PWB) in the workplace has recently been spotlighted as a way to improve organizational performance, by promoting the mental health of employees. This is because, when employees perceive themselves to be mentally healthy, there can be fewer negative factors such as stress, burnout syndrome, and exhaustion that occur in the work process. Workers can achieve self-actualization through PWB [9], and such psychological satisfaction increases when they give a great amount of meaning to their careers by themselves or have a sense of calling [6]. As such, it is necessary for managers/executives to focus on PWB in the workplace because psychological satisfaction enables employees to show/develop their potential [10].

In order to explain the behavior of individuals in an organization, the relationship between the contextual attributes of the organization and the characteristics of the individuals is very important [11]. In addition, organizational culture is a general term for the values, beliefs, ideology, customs, etc., of the members of the organization, and has a great influence on the organization and its members. It has been found in several studies that the self-directed tendencies (personal values) of employees form the culture within the organization, and that this culture influences the performance of the organization. Therefore, it can be seen that research is needed to improve mental health within the organization by forming an organizational culture through self-directed career management.

From this point of view, this study verified the dual mediation effects of career satisfaction and career commitment in the relationship between PCA and PWB. Most previous studies on PCA focused on subjective career success, organizational effectiveness, and organizational performance as outcome factors of PCA; there has been a lack of studies on PWB within organizations, and on mediating roles of career satisfaction and career commitment. In this sense, this study focused on PCA, which has been recently spotlighted due to its emphasis on individual self-directedness, it analyzed the impact on PWB through the dual mediation effects of career satisfaction and career commitment, and it attempted to identify how the mediation effects were presented in the relationship. Through this process, this study aimed to expand the theoretical scope of PCA and investigate the effect on mental health in the workplace. Based on these research results, it provides theoretical and practical implications, and, eventually, practical insight into sustainable business management methods via PCA. This article consists of six sections: Introduction, Literature Review and Hypotheses, Study Method, Results, Discussion, and Conclusions.

## 2. Literature Review and Hypotheses

### 2.1. Protean Career Attitude and Career Satisfaction

A career is the sum of experiences that encompass work-related activities, values, and expectations that persist throughout an individual’s lifetime [12]. In the past, career development focused on improving the competencies of organizational members according to career paths designed by organizations, while considering long-term employment relationships [13]. However, due to the recent rapid socioeconomic changes and the emergence of a flexible labor market, the concept of a lifetime workplace is gradually disappearing. Traditional career management focused on promotion, salary, and stability, and was organization-oriented, but a new paradigm of career management highlighting individual psychological success and values is emerging [5].

Briscoe and Hall [14] classified an individual’s career orientation into four types (i.e., dependent, rigid, reactive, and protean types) based on the combination of self-directed attitude and value-driven attitude. Among them, PCA is a concept that represents career management in a changing environment from traditional career management that pursues subjective psychological success and manages careers independently [3,5,6,8]; it is a career management/development that involves the entire personal life managed by an individual [15,16].

PCA was introduced by Hall [5], and it is a career development by an individual, not an organization, and aims at personal-psychological success [5]; it is career development in which an individual changes his/her work, organization, and occupation type to meet his/her demand, and it is re-formed through the individual’s career choice and efforts for self-fulfillment [1]; it is also created by continuous learning, individual value, and purpose of life [1,5,8]. Through PCA, individuals emphasize personal values for career success and have self-directed and value-driven features in career management, such as work performance and learning [14].

The sub-dimensions for PCA consist of self-directed and value-driven subfactors [3,14,17]. The first sub-dimension is self-directed (SD). SD refers to the degree to which individuals try to take a leading role in developing and managing their careers, rather than relying on external objects [14]. Through SD, individuals manage their careers and build network relationships so that they can create opportunities for growth through active task performance [3,14]. The second sub-concept, value-driven (VD), is related to enhanced satisfaction and commitment by placing more importance on one’s own values when making career-related decisions [14,15].

PCA is closely related to the improved performance of a company’s employees. Individuals with PCA are proficient in professional development and work-related networking [15], show a tendency to plan career goals based on personal values and psychological standards [3], and are active in self-management and self-development [18].

In particular, PCA enables employees to grow through new experiences and learning processes [6] and change individual interests, abilities, and values in organizational contexts [7]. Furthermore, in the recently rapidly changing business environment, employees with higher PCA give high value to internal success and have a high spirit of challenge and desire for growth.

In other words, the core value of the PCA lies in psychological success such as subjective satisfaction and achievement and focuses on personal psychological growth. Therefore, PCA’s core values focus on freedom and personal growth, and the value of a career managed by an individual is judged by subjective values; it has a close relationship with subjective success and happiness in measuring personal-psychological success and satisfaction. Therefore, companies should focus on PCA, and in-depth studies on the effects and impacts of PCA are required.

As such, PCA is important to respond effectively to changes in the career environment. Therefore, among the two sub-dimensions of PCA, self-directedness can be seen as the capacity to change proactively with environmental changes. Therefore, this study aims to analyze the self-direction of PCA, which enables individuals to take charge of their career management for high corporate performance.

On the other hand, career success refers to an accumulated positive psychological state due to personal work experiences or work-related outputs [19]. Career success is an accumulated positive psychological state due to individual work experiences, and was mainly evaluated by objective criteria such as wages, positions, and social status in the past [20]. Therefore, it can be considered an accumulated positive psychological state due to personal work experiences or work-related outputs [21].

Subjective career success is a personal perception of one’s career, job, career achievement, and career satisfaction [22,23], and is a more complex and multi-dimensional concept than objective career success [24]; it can be defined as an individual’s subjective judgment and evaluation of their careers [20]. That is, careers determined by individual subjectivity include individual career-related factors such as career achievement, career pleasure, and career satisfaction [20]. Furthermore, since psychological success such as personal achievement and satisfaction is included, it is discussed as a more comprehensive concept than objective career success [25].

Career satisfaction (CS), a representative concept of subjective career success, refers to the level to which an individual’s value is applied to one’s career [26]. CS is an attitude towards one’s career and can be considered the degree to which one psychologically identifies with one’s career and perceives oneself as successful in one’s field [22]. CS refers to individuals’ overall psychological orientation toward their own careers [24], and satisfaction with their achievement level in their career goals, wages, promotion, and development, as successes in their career [27].

Since satisfaction with one’s career is the degree to which individuals perceive that they have been successful in their career, it can be seen that the higher the career satisfaction, the more immersed in one’s career activities and work they are [28]. Therefore, CS can be considered an important indicator for measuring career success, and is the most widely used measurement.

Individuals with PCA can be motivated by improving their abilities and challenging their goals through a great amount of learning and development [17]. PCA is also closely related to CS because one takes a lead in one’s career management based on individual subjective judgment [6]. This is because, as the career progresses, the individual’s subjective sense of satisfaction becomes more important because the career is evaluated according to one’s own values and beliefs [15]. Therefore, it can be seen that the higher the PCA level, the more value-driven and self-directed is their career management; there is a tendency to be satisfied with their career or job. This is because PCA pursues psychological success through career management led by individuals [14], and career success is determined by putting more value on psychological satisfaction [29]. Therefore, it can be seen that PCA and CS have a major relationship.

As for previous studies regarding PCA and CS, De Vos and Soens [7] conducted an empirical study on 274 Belgian employees and found that PCA had a positive impact on CS and employability via career insight in the structural relationship between PCA, career management, and career success. In addition, PCA was found to have a full mediation effect for career insight on CS and perceived employability, emphasizing that PCA was an important prerequisite for CS. Enache et al. [30] conducted a study on 150 Spanish graduate students and indicated that the SD aspect of PCA had a significant positive impact on CS. Herrmann et al. [31] showed that PCA enhanced active career behavior and CS through the active tendency. As such, several previous studies indicated that the higher level of self-directed career management via PCA led to a positive impact on CS.

Therefore, it is worth pursuing an examination of the effect of PCA on CS, and we propose the following hypothesis:

**Hypothesis** **1** **(H1).** *Protean career attitude positively influences career satisfaction.*

### 2.2. Protean Career Attitude and Career Commitment

Due to the recent organizational environment changes and the expansion of employment flexibility, career commitment (CC) is crucial for proactive career goal achievement and sustainable career management [32,33]. CC is the level of motivation to work in one’s chosen occupation [34] and is a representative concept for measuring career performance.

CC indicates one’s commitment to one’s occupation (career), rather than one’s organization [35], and has greater relevance to tasks related to one’s career; one can achieve high performance through this [36]. CC can play an important role in maximizing individual capabilities and maintaining attachment to one’s professional field [37], improving the professionalism and competencies of members, and increasing organizational performance [38]. From the organizational point of view, employees with higher CC improve organizational performance because they keep in mind the goals and values of the organization [38]. Effective management of the organization is possible because it is possible to efficiently maintain and manage manpower [34]. As such, CC is a crucial factor for maintaining individuals’ careers in the labor market and enables them to maintain their careers by being motivated and committed to achieving individual career goals [33].

Leading career management by placing more importance on core concepts of PCA is crucial for personal career growth [26]; if the value is given to a career, CC can be improved and one can achieve higher performance [39]. There is a deep relationship between PCA and CC because people with self-directed career attitudes become committed to their careers while trying to achieve their career goals [40].

As for previous studies related to PCA and CS, Noor et al. [41] conducted a study on 267 Indonesian civil servants and showed that career commitment increased through self-directed career management behavior, emphasizing the importance of career management. De Vos et al. [42] targeted 491 employees of large companies and indicated that self-directed career management had a positive relationship with commitment and career success.

Combining the abovementioned previous studies, it can be expected that there was a positive impact on CC when organizational members had higher PCA and managed their careers in a self-directed manner. This is because one can have motivation for their job when the organization’s job matches the career value pursued by an individual [43], and as they actively work on their job, the sense of responsibility and achievement is improved, leading them to commit to their careers [32]. Therefore, this study expected that PCA would have a positive effect on CC, and the following hypothesis was established:

**Hypothesis** **2** **(H2).** *Protean career attitude positively influences career commitment.*

### 2.3. Protean Career Attitude and Psychological Well-Being

When it comes to promoting the mental health of members, psychological well-being (PWB) has recently attracted attention as a method to improve organizational performance. PWB indicates self-realization (eudaimonia) that is achieved through the process of discovering the satisfaction and potential that an individual experiences through life [9], and is a process of finding oneself as a social being and getting closer to true happiness (i.e., Being Good) [44]. PWB is a state in which organizational members discover their potential as well as psychological satisfaction in life [11], encompassing the concepts of mental health, beliefs, quality of life, and well-being [45], and is being studied to measure an individual’s state of life and psychological satisfaction [46].

PWB is the realization of an individual’s potential and is closely related to growth and development, challenges, and efforts in life [9,11]. Furthermore, it can appear as a result of not only an individual’s positive response to surrounding environments, but also an interaction between organizational environments that individuals experience such as the organization’s system, superiors, colleagues, and each member of the organization [47].

Individuals with positive emotions show a creative disposition for their situation, challenge new goals with an active mindset, and can achieve higher performance in the process [48]. In addition, employees with PWB can respond more effectively to the roles required by their organizations, secure psychological resources to overcome burnout [49], and achieve higher performance as there is an impact on work performance, not just obtaining satisfaction and happiness through work [50]. Therefore, PWB is an important factor in corporate and organizational management.

In the relationship between PCA and PWB, members with PCA aim for subjective psychological success [6]. Since PCA aims to achieve psychological success, such as a sense of accomplishment, family happiness, and stability, rather than salary increases or promotion in a vertical corporate system [5], there is a deep connection between them.

Briscoe et al. [51] found that the ‘self-directed’ dimension of PCA had a positive impact on and a significant relationship with PWB. Li [52] indicated the effect of PCA of knowledge workers on PWB via psychological capital and showed that PCA had a positive impact on PWB, and psychological capital had a partial mediation effect. Rahim and Siti-Rohaida [53] conducted a study on Malaysian engineers and found that PCA had a positive effect on PWB via Career Goal Development. Mustafa et al. [54] showed that individuals with higher PCA had higher self-efficacy, hopeful states, and more positive mindsets for career success.

Based on the above-mentioned studies, it was found that PCA had a significant relationship with PWB, so as to help a company develop into a company with sustainable high-performance in a changing era, beyond the higher corporate survivability. PWB can be regarded as an individual success for corporate growth and development, and individual and organizational growth can be expected by achieving individual goals. Thus, we advance the following hypothesis:

**Hypothesis** **3** **(H3).** *Protean career attitude positively influences psychological well-being.*

### 2.4. The Relationship between Career Satisfaction, Career Commitment, and Psychological Well-Being

As individuals have higher career satisfaction, they are more committed to their jobs (careers) than their organizations [35]; as there are greater associations with tasks related to their careers, they can achieve higher performance. Therefore, CS and CC are closely related [36]. As they give more meaning to their careers and they have a sense of calling, their career satisfaction and career commitment can become higher [6]. Furthermore, CC appears according to the level of competence, success, and perception within the current career role [15], allowing them to experience emotional attachment in their current career and achieve higher performance [29].

From an organizational perspective, if employees are satisfied with the careers that they have accumulated while performing successful tasks in their field, and if they commit to their careers and obtain psychological satisfaction, they can achieve higher organizational performance because job performance is improved [55].

Numerous previous studies indicated that CS and CC were factors that improved PWB. Xu et al. [56] targeted 213 full-time medical workers in public hospitals in southern China and found that supervisory career support and CC had a positive impact on PWB. Haar and Brougham [57] targeted 172 Maori workers in New Zealand and indicated that CS had a positive effect on PWB.

In addition, CS and CC were found to have a strong correlation. Aryee et al. [29] verified the CS-related model and found that CS directly affected CC. Tak and Lim [27] targeted IT company employees and examined the relationships between full-time, temporary, professional, and non-professional staff, indicating that CS had a positive impact on CC. Onyishi et al. [58] targeted 233 Nigerian public hospital nurses and examined the impact of psychological needs satisfaction on CC via CS, finding that psychological needs satisfaction had a positive impact on CS and CC and that psychological needs satisfaction had a significant impact on CC through CS; they emphasized the importance of mental health in career management in the workplace.

Combining the discussed previous studies, it can be expected that the relationship between CS and CC, and with PWB will provide a positive relationship. Thus, we advance the following hypotheses:

**Hypothesis** **4** **(H4).** *Career satisfaction positively influences psychological well-being.*

**Hypothesis** **5** **(H5).** *Career commitment positively influences psychological well-being.*

**Hypothesis** **6** **(H6).** *Career satisfaction positively influences career commitment.*

### 2.5. Mediation Effect of Career Satisfaction, Career Commitment

PCA puts more emphasis on subjective elements such as psychological success (e.g., an individual’s sense of purpose in life) rather than objective successes such as wages, position, and power [14]. In other words, it refers to measuring psychological career success based on an individual’s subjective criteria, placing more value on subjective cognitive aspects rather than visible success [29]. In the study on the relationship between CS and CC, and with PWB, CS and CC are factors that improve organizational performance by systematizing the management system within the organization. In particular, self-directed career management is an important antecedent factor closely related to CS and CC.

As for previous studies, Zulkarnain [59] showed that the quality of work-life had a very deep correlation with the relationship between career development behavior and PWB. Xu et al. [56] targeted 213 full-time medical workers in public hospitals in southern China and found that CC had a significant mediation effect on the relationship between supervisory career support and PWB. Joo and Lee [60] targeted 550 employees of large Korean companies and found that psychological capital had a full mediation effect on wellbeing through career satisfaction. Aggarwal-Gupta and Vatharkar [61] targeted 102 physicians and indicated that job satisfaction and CS fully mediated the relationship between perceived stress and mental well-being, highlighting the importance of job satisfaction and career satisfaction to ensure the mental health of physicians.

Based on those previous studies, it was found that PCA had a positive effect on CS and CC because it is related to self-directed career management [7,40,62,63]; CS and CC were found to have a positive impact on psychological satisfaction and PWB. One of the main proposals of this study is that PCA has a significant effect on PWB. However, there are few studies on the mediation effects of CS and CC in the relationship between PCA and PWB. Based on the results of several previous studies, it is highly likely that there is a deep relationship with individual PWB, since career satisfaction and career commitment are emotional and psychological products of individuals. Therefore, this study expected that CS and CC would have a positive mediation effect on the relationship between PCA and PWB, and hypotheses were established as follows:

**Hypothesis** **7** **(H7).** *Career satisfaction mediates the relationship between protean career attitude and psychological well-being.*

**Hypothesis** **8** **(H8).** *Career commitment mediates the relationship between protean career attitude and psychological well-being.*

There may be various mediating factors in the structural relationship between PCA and PWB. Those previous studies indicated that CS and CC played a role as parameters or dependent variables. In fact, since there are correlations between parameters in various research models, it is also important to analyze the causal relationship [64,65].

Since PCA is related to self-directed and value-driven career management, it can lead to career satisfaction and career commitment. Furthermore, psychological satisfaction obtained through eudaimonia via career management [9] is highly likely to function as promoting PWB. Therefore, this study attempted to elaborately analyze the structural relationship between PCA and PWB by examining the dual mediation effect of CS and CC.

**Hypothesis** **9** **(H9).** *Career satisfaction and career commitment dual mediate the relationship between protean career attitude and psychological well-being.*

### 2.6. The Hypothesized Research Model

**Figure 1:**
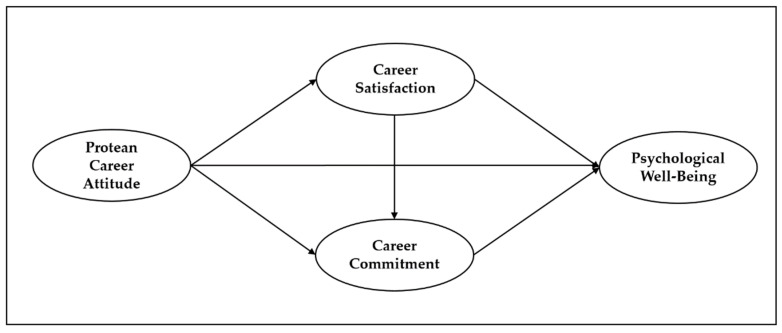
Hypothesized research model.

The relationships between the important variables under examination are shown in the research model in Figure 1. This study made the assumption that a protean career attitude impact on psychological well-being is real. It developed career satisfaction and career commitment as parameters to illustrate such a system in concrete terms.

## 3. Study Method

### 3.1. Sample and Procedures

Many companies need to make efforts to deal with changes brought on by the recent Fourth Industrial Revolution and the rapidly changing COVID-19 environment, in order to survive as sustainable companies. It is meaningful to verify the effectiveness of PCA, particularly for Korean small and medium-sized enterprises (SMEs), which are sensitive to such aforementioned changes. Therefore, a survey for this study was conducted on full-time workers of small and medium-sized enterprises (SMEs) in South Korea in January 2022. The questionnaires were initially sent by email to 330 workers.

However, the sample was limited to those aged 25 to 59 years who were graduates from high school or college and who had worked for one year or more in the workplace. The final sample was reduced to 307 employees after we further deleted 23 cases because there were too many crucial variables that had received no response (93.3%).

We limited the sample to those who were 25 years old or older and who had more than one year of work experience. This is because it is difficult to measure the relevant items when the number of years of experience is too low.

### 3.2. Measures

We prepared the questionnaire after it was reviewed by experts in the relevant fields and after a literature review of previous studies. The questionnaire was proofread by native speakers living in Korea so that it would convey the exact meanings to the respondents.

Scales for each variable were previously validated by study. All assessment items had a response format of a Likert-type scale with a range of 1 (strongly disagree) to 5 (strongly agree).

#### 3.2.1. Protean Career Attitude (PCA)

To measure protean career attitude, we adapted Briscoe, Hall, and DeMuth’s [3] scales.

Protean career attitude (self-directed) was measured by three items and sample items included the following: “Overall, I have a very independent and self-directed career.”

#### 3.2.2. Career Satisfaction (CS)

Career satisfaction was assessed by a four-item scale adapted from Greenhaus et al. (1990) [12]. The sample items included: “Looking back on my career, I am satisfied with the current successes (performances)”.

#### 3.2.3. Career Commitment (CC)

Career commitment had a three-item scale (Cronbach’s α = 0.86) adapted from Blau (1985) [35]. Blau (1985) developed a measurement tool considering career commitment as a concept in which individuals can commit to their jobs, and develop and manage their careers to achieve higher performance, rather than committing to their organizations (i.e., organizational commitment) [35]. The sample item was: “I have a high attachment to my current career.”

#### 3.2.4. Psychological Well-Being (PWB)

Psychological well-being was measured by using six items (Cronbach’s α = 0.69) adapted from Ryff and Keyes (1995) [9]. The sample question was: “I give meaning to my life and I live it while feeling worth it.”

### 3.3. Statistical Analysis

The structural equation model (SEM) was used in this study to verify the proposed research hypotheses based on the theoretical background. The general characteristics of the sample were analyzed by performing demographic statistical analysis on the collected dataset using SPSS 25. Confirmatory factor analysis and reliability tests were performed using AMOS 22 and SPSS 25 in order to test the proposed hypotheses based on the theoretical background.

Each proposed hypothesis was then tested using SEM, and the phantom model method was used to measure the indirect effect, in order to verify the indirect effect of the research model.

### 3.4. Institutional Review Board (IRB) Approval

The survey was conducted on human subjects for the study in accordance with the guidelines of the Declaration of Helsinki regarding human research studies, and it was carried out according to the guidelines of P0120220201006 IRB after being reviewed by the Public Institutions Review Committee of the Republic of Korea.

## 4. Analysis Results

### 4.1. Information of Survey Participants

In terms of gender, the respondents included 185 males (60.3%) and 122 females (39.7%); 60 respondents were aged between 25 and 29 years (19.5%), 129 between 30 and 39 years (42.0%), 92 between 40 and 49 years (30.0%), and 26 were between 50 and 59 years (8.5%); 32 respondents were high school graduates (10.4%), 47 had been undergraduates (15.3%), and 228 were college graduates or had higher academic levels (74.3%).

Regarding the type of occupation, 218 were office workers (71.0%), 17 were sales workers (5.5%), 32 were R&D workers (10.4%), 27 were production line workers (8.8%), and 13 were in other job categories (4.2%). As for social career experience, 33 people (10.7%) with 1 to 3 years, 47 people (15.3%) with 4 to 6 years, 72 people (23.5%) with 7 to 10 years, and 155 people (50.5%) with 11 or more years were surveyed. Based on the current company size (workforce), 145 respondents (47.2%) worked in companies with 10–50 full-time employees (including unlimited contract employees), 75 (24.4%) in companies with 51–150 full-time employees, 87 (26.9%) in companies with at least 151 full-time employees, and the majority of respondents worked in small and medium-sized enterprises with up to 150 full-time employees.

### 4.2. Confirmatory Factor Analysis

We first performed series-wise confirmatory factor analysis to examine the distinctiveness of the scales protean career attitude, career satisfaction, career commitment, and psychological well-being using Amos Version 22 and SPSS Version 25.

The proposed four-factor model exhibits a better fit to the data than all alternatives, as seen in Table 1, which is published RMSEA = 0.068; CFI = 0.951; NFI = 0.920; χ^2^/*df* = 2.411, *p* < 0.001. The results of the analysis show that the four-factor model is the most suitable for this research.

The findings of CFA used to assess the composite reliability and convergent validity of the four-factor model are presented in Table 2. The measurement model’s goodness of fit had the following reference values: RMR = 0.034, GFI = 0.912, NFI = 0.920, IFI = 0.952, TLI = 0.940, CFI = 0.951, RMSEA = 0.068. CFI, GFI, NFI, IFI, TLI, and GFI all exceeded the cutoff (>0.9), indicating satisfactory model fit.

The convergent validity of the measurement model was also examined, and the analytical findings showed that all of the observed variables had factor loadings that were acceptable (λ > 0.5) [66]. Additionally, the critical ratio (C.R.) value, signifying significance (*p* < 0.001), was above the 1.965 reference value, confirming that the value was significant.

Additionally, it was determined that the average variance extracted (AVE > 0.5) and composite reliability (CR > 0.7) both met the requirements for convergent validity.

Similarly, Cronbach’s coefficient alpha and composite reliability were used to test the construct-level reliability or convergent validity. The findings demonstrate that Cronbach’s alphas exceeded the threshold cutoff of 0.60.

As a result, the model of the research model proposed in this study was found to be suitable through the confirmatory factor analysis of Table 1 and Table 2.

Correlations between the study variables are shown in Table 3. By comparing the square value of the correlation coefficient between the components, discriminant validity was established [66]. As shown in Table 3, the largest value of the correlation coefficient between variables was 0.706 (the relationship between career satisfaction and career commitment), and the smallest value among the square roots of the AVE value was 0.708 (protean career attitude). Therefore, it was found that the discriminant validity of the proposed model is satisfied.

### 4.3. Test of Hypotheses

The findings from the analysis of the hypothesized relationships using the Structural Equation Modeling (SEM) method are presented in Table 4. The model fit was confirmed by structural model analysis to be satisfactory (CFI = 0.951, TLI = 0.940, IFI = 0.952, RMR = 0.034, RMSEA = 0.068).

Hypothesis 1 predicted that protean career attitude will positively influence career satisfaction. In Table 4, protean career attitude was found to have a significant effect (β = 0.491, *p* < 0.001) on career satisfaction. Therefore, Hypothesis 1 that protean career attitude has a positive (+) effect on career satisfaction was supported.

Hypothesis 2 of this study predicted that PCA would have a positive effect on career commitment. Hypothesis 2 was supported, as it was found that PCA had a significant effect (β = 0.287, *p* < 0.01) on career commitment. Thus, Hypothesis 2 was supported. In the verification of Hypothesis 3 that PCA had a positive effect on PWB, PCA was found to have a significant effect (β = 0.366, *p* < 0.001) on PWB. Therefore, Hypothesis 3 was supported.

Hypotheses 4 and 5 are the relationships between the mediator variable used in this study (career satisfaction and career commitment) and psychological well-being. Hypothesis 4 that career satisfaction has a significant effect on psychological well-being was not supported (β = 0.102, *p* = n.s.). However, Hypothesis 5 that career commitment affects psychological well-being was found to be significant (β = 0.361, *p* < 0.001).

In the verification of Hypothesis 6 on the relationship between the two mediator variables used in this study, the effect of career satisfaction on career commitment was significant (β = 0.565, *p* < 0.001). Thus, while Hypotheses 5 and 6 were supported, Hypothesis 4 was not supported.

Table 5 shows the results using a bootstrap bias-correction approach with 2000 samples taken to investigate Hypothesis 7–9 that career satisfaction and career commitment act as parameters between protean career attitude and psychological well-being.

Hypothesis 7 predicted that career satisfaction, the first parameter of this model, would play a significant mediation role in the relationship between PCA and PWB. However, the indirect effect of CC on the relationship between PCA and PWB was not significant (estimate = 0.053, *p* = n.s.). Therefore, Hypothesis 7 was rejected. In the verification of Hypothesis 8, in which career commitment, the second parameter of this study, would mediate the relationship between PCA and PWB, it was shown that career commitment significantly mediated the relationship between PCA and PWB (estimate = 0.109, *p* < 0.05). Therefore, Hypothesis 8 was supported.

In the verification of Hypothesis 9 to measure the dual mediating effect on the relationship between protean career attitude and psychological well-being, PCA showed a dual mediating effect on psychological well-being through career satisfaction and career commitment (estimate = 0.106, *p* < 0.05). Furthermore, the total indirect effect was also significant (estimate = 0.267, *p* < 0.01). Therefore, Hypothesis 9 was supported.

## 5. Discussion

In this article we explored the linkage of protean career attitude, characterized as self-directed and value-driven, to psychological well-being.

To this end, we used career satisfaction and career commitment as mediators in the research model. The findings of our study provided evidence for the proposed hypotheses, but some hypotheses were not supported.

The results of this study are as follows. First, PCA was found to have a positive effect on career satisfaction, and PCA was found to have a positive effect on career commitment.

Second, PCA was found to have a significant effect on PWB. This result supports the study of Rahim and Siti-Rohaida [53], and confirms that self-directed career development was appropriate for promoting PWB in the workplace.

Third, career satisfaction was found to positively affect career commitment, and career commitment was found to have a significant impact on PWB. Fourth, as a result of verifying the double mediation effects of career satisfaction and career commitment, which are structural models of this study, it was found that protean career attitude improved psychological well-being through career satisfaction and career commitment.

### 5.1. Theoretical and Practical Implications

Through this process, we derived the following implications. First, this study verified the structural relationship in which PCA can improve corporate performance by enhancing the PWB of employees in the workplace through career satisfaction and career commitment. In most previous studies, PCA has been mainly studied as a mediating model through a single variable. However, this study has theoretical implications in that it expanded the scope of existing studies by suggesting PCA, career management behavior, and a mechanism for a new structural relationship differentiated from previous studies on PCA, through an analysis of dual mediation effects. It is also significant in terms of statistical and methodological aspects, as it verified the relationship through a structural equation model via bootstrapping.

Second, PCA improved an individual’s career satisfaction, and PCA appeared to improve career commitment and PWB. As had been indicated in previous studies, it was reverified that career commitment within an organization contributed to improving various financial and non-financial performances. Although career satisfaction did not have a significant effect on PWB, an indirect effect can be expected from the effect relationship. Therefore, it can be seen that career performance had a positive effect on the PWB of employees in the workplace.

From these results, companies should support their employees to become self-directed through education and training programs, such as various special lectures and seminars, so that they can develop their careers independently. If there are efforts to improve career performance, it will be effective in promoting the PWB of members. Furthermore, the theoretical scope was expanded by demonstrating that corporate performance can be improved through this process.

Third, career satisfaction was found to have a positive effect on career commitment. It was also found that career commitment positively affected PWB. This is also related to the structural model of this study. In the dual mediation effect through PCA, PCA was found to improve PWB through career satisfaction and career commitment, reconfirming the importance of self-directed career development for improved PWB in the workplace. Therefore, companies should support their employees to actively perform tasks, while employees manage their careers independently.

Fourth, the research scope was expanded through the analysis of the causal relationship between PCA and mental health in the workplace; the causal relationship that PCA increased PWB in the workplace was found through the mediation effects of career satisfaction and career commitment. Based on these results, this study suggested the importance of PCA in individual career management, and as the basis for sustainable corporate management through the improved mental health of employees in the workplace.

Finally, as this study suggests the importance of PCA to promote mental health in organizations, it can be considered that this study made a major contribution to the career management field. In particular, during a period of rapid industry changes, companies should pay attention to the stress levels of workers due to department change or turnover. As such, if the management recognizes the importance of PCA, and of employees’ career satisfaction and career commitment, this will contribute to improving the working environments of companies, and the companies will be able to achieve sustainable high-performances.

### 5.2. Limitations and Future Research Directions

Although this study academically and practically contributed to understanding the relationship between PCA and PWB, it is necessary to focus on the interpretation of the results, because there are a few limitations. There should be follow-up studies that supplement these limitations in the future.

First, this study empirically analyzed the effects of career commitment and career satisfaction in the relationship between PCA and PWB in the workplace and provided implications for the results. However, in analyzing the structural relationship through the research model of this study, it is necessary to study the causal relationship through a more specific research design for variables that appear insignificant in the future.

Second, this study was conducted during a global health crisis, the COVID-19 pandemic. It is necessary to be careful with interpretation during this period because of mental stress exacerbated by passive management, driven by economic contraction, and the implementation of a social distancing system.

Third, there is a regional limitation in that this study targeted only South Korea. Therefore, it is suggested that future research should include more countries. The impact of PCA on PWB in the workplace may differ depending on companies’ values or goals, ethnicity, or culture. Therefore, future studies should reflect various cultures.

Fourth, this study verified the hypotheses by using the same measurement tool from a single respondent through a survey of the subjects. This implies a common method bias problem, which may distort the research results due to a lack of self-awareness. Therefore, improvements through multiple questionnaires or time series analysis are needed in future studies.

Fifth, the study analyzed the self-directed attitude of PCA as a study criterion. PCA has two sub-dimensions of self-directed and value-driven attitudes. Studies on the scale of PCA are still lacking; instead, there are several studies on diagnostic tools, such as a study conducted by Briscoe showing that some items have significant cross-loading for Self-directed and Value-driven. Therefore, this study suggests that exploratory factor analysis of PCA-related items and various studies analyzing all factors of PCA need to be conducted in the future.

In addition, since this study is cross-sectional, it is necessary to strengthen the causal relationship between each variable through a longitudinal study in future studies. Therefore, it is necessary to build a more extensive model.

## 6. Conclusions

In a changing business environment, protean career attitudes and the promotion of psychological well-being in the workplace are the driving forces for improving corporate performance and achieving sustainable growth. In this context, protean career attitudes and psychological well-being are emphasized. This study makes an important contribution to the field by pointing out that the protean career attitude of self-directed career management increases psychological well-being in the workplace. In particular, this study examined the structural relationship between them by verifying the double mediating effect of career satisfaction and career commitment in this relationship.

An important point is that PCA, which manages careers in a self-directed fashion, improves psychological well-being in the workplace through career satisfaction and career commitment. In particular, this point highlights the importance of PCA for managers and middle managers, particularly in the recent management trend of improving the quality of life and psychological well-being of horizontal organizational culture. In conclusion, PCA plays an important role in the sustainability of SMEs greatly affected by the rapidly changing business environment by improving mental health in the workplace.

## Figures and Tables

**Table 1:** Confirmatory factor analysis.

Model	Description	χ^2^	*df*	χ^2^/*df*	CFI	NFI	RMSEA	RMR	Change from Model 3	
									Δ χ^2^	Δ *df*
1	One-factor model ^a^	247.790	100	2.478	0.948	0.916	0.069	0.041	11.506	2
2	Three-factor model ^b^	236.399	99	2.388	0.952	0.920	0.067	0.034	0.115	1
3	Four-factor model ^c^	236.284	98	2.411	0.951	0.920	0.068	0.034		

N = 307. CFI = Comparative fit index; NFI = Normed fit index; RMSEA = Root mean square error of approximation; RMR = Root mean square residual. ^a^ Protean career attitude, career satisfaction, career commitment, and psychological well-being combined together as one construct. ^b^ Two mediators (career satisfaction and career commitment) combined as one construct with protean career attitude and psychological well-being as separate constructs. ^c^ Hypothesized model in which all items are separate constructs.

**Table 2:** Results of the confirmatory factor analysis of the four-factor model.

Latent Variable		Estimate	C.R.	Standardized Estimate (λ)	Cronbach’s α	AVE	Composite Reliability
Protean career attitude	PCA1	1	-	0.724 ***	0.749	0.501	0.751
	PCA2	0.846	9.950	0.705 ***			
	PCA3	0.874	9.858	0.694 ***			
Career satisfaction	CS1	1	-	0.833 ***	0.871	0.630	0.871
	CS2	1.068	17.613	0.874 ***			
	CS3	1.003	14.256	0.740 ***			
	CS4	0.961	13.654	0.716 ***			
Career commitment	CC1	1	-	0.776 ***	0.839	0.635	0.839
	CC2	1.080	14.114	0.817 ***			
	CC3	1.031	13.811	0.797 ***			
Psychological well-being	PWB1	1	-	0.769 ***	0.915	0.644	0.915
	PWB2	1.101	16.114	0.858 ***			
	PWB3	1.093	15.109	0.813 ***			
	PWB4	1.022	15.218	0.818 ***			
	PWB5	1.030	15.088	0.812 ***			
	PWB6	1.007	13.503	0.740 ***			

Model fit: χ^2^/*df* = 2.411 (*p* < 0.001), RMR = 0.034, GFI = 0.912, NFI = 0.920, IFI = 0.952, TLI = 0.940, CFI = 0.951, RMSEA = 0.068. C.R.: critical ratio, AVE: average variance extracted. *** *p* < 0.001.

**Table 3:** Correlation and with the square roots of the average variation extracted at the diagonal.

Variables	1	2	3	4
1. Protean career attitude	**0.708**			
2. Career satisfaction	0.491 **	**0.794**		
3. Career commitment	0.564 **	0.706 **	**0.797**	
4. Psychological well-being	0.620 **	0.536 **	0.640 **	**0.802**

Note: square roots of AVE in bold at the diagonal. ** *p* < 0.01.

**Table 4:** Results of SEM (Structural Equation Modeling).

Hypotheses				Estimate	S.E.	β	Result
H1	Protean career attitude	→	Career satisfaction	0.549	0.083	0.491 ***	Supported
H2	Protean career attitude	→	Career commitment	0.307	0.075	0.287 ***	Supported
H3	Protean career attitude	→	Psychological well-being	0.386	0.081	0.366 ***	Supported
H4	Careersatisfaction	→	Psychological well-being	0.096	0.075	0.102	Rejected
H5	Career commitment	→	Psychological well-being	0.356	0.090	0.361 ***	Supported
H6	Careersatisfaction	→	Career commitment	0.541	0.067	0.565 ***	Supported

Model fit: χ^2^/*df* = 2.411 (*p* < 0.001), RMR = 0.034, GFI = 0.912, NFI = 0.920, IFI = 0.952, TLI = 0.940, CFI = 0.951, RMSEA = 0.068. S.E.: Standard error, β: standardized coefficients. *** *p* < 0.001.

**Table 5:** Results of indirect effect analysis of the proposed model.

Relationship of Variables		Indirect Effect	Lower	Upper	Result
	Indirect Effect				
H7	PCA→CS→PWB	0.053	−0.048	0.153	Rejected
H8	PCA→CC→PWB	0.109 *	0.030	0.223	Supported
H9	PCA→CS→CC→PWB	0.106 *	0.039	0.200	Supported
	Total Indirect Effect	0.267 **	0.149	0.422	-
	Direct Effect	0.386 *	0.178	0.557	-
	Total Effect	0.653 **	0.518	0.790	-

PCA: Protean career attitude, CS: Career satisfaction, CC: Career commitment, PWB: Psychological well-being. ** *p* < 0.01, * *p* < 0.05.

## Data Availability

Not applicable.
